# When Flow Voids Are Missed: Delayed Diagnosis of Foix–Alajouanine Syndrome—A Case Report

**DOI:** 10.3390/neurolint18100188

**Published:** 2026-10-07

**Authors:** Dominika Jakubowicz-Lachowska, Mateusz Wierciński, Katarzyna Kapica-Topczewska, Alina Kułakowska, Eugeniusz Tarasow, Jan Kochanowicz, Monika Chorąży

**Affiliations:** 1 Department of Neurology, Medical University of Białystok, 15-276 Białystok, Poland; 2 Department of Radiology, Medical University of Białystok, 15-276 Białystok, Poland

**Keywords:** Foix–Alajouanine syndrome, spinal dural arteriovenous fistula, perimedullary flow voids, congestive myelopathy, magnetic resonance imaging, digital subtraction angiography, diagnostic delay

## Abstract

**Introduction:** Foix–Alajouanine syndrome (FAS) is a rare, progressive congestive ischaemic myelopathy caused by a spinal dural arteriovenous fistula (SDAVF). Because of its non-specific clinical presentation and subtle imaging features, FAS is frequently misdiagnosed; in particular, perimedullary flow voids—its characteristic imaging hallmark—are readily overlooked on initial MRI, leading to diagnostic delay and potentially harmful interventions. **Case Presentation:** We report the case of a 66-year-old man who developed progressive myelopathy following revision knee arthroplasty performed under spinal anaesthesia. Initial MRI at another institution demonstrated extensive intramedullary T2 hyperintensity, interpreted as spinal cord infarction, while perimedullary flow voids were present but overlooked. Lumbar puncture and high-dose corticosteroid therapy were followed by marked neurological deterioration. At our centre, re-evaluation of a 3T MRI revealed serpentine perimedullary flow voids, and digital subtraction angiography confirmed an SDAVF at the Th5–Th6 level. **Outcome:** Transarterial endovascular embolisation with Onyx resulted in neurological stabilisation and partial recovery, with reduced pain and improved bladder control at follow-up. **Conclusions:** This case highlights the importance of recognising perimedullary flow voids, which may otherwise lead to delayed diagnosis of SDAVF. In patients with unexplained longitudinally extensive myelopathy, particularly when the clinical course is atypical or worsens after lumbar puncture or corticosteroid therapy, the MRI should be carefully reviewed for flow voids and dedicated spinal vascular imaging considered.

## 1. Introduction

Foix–Alajouanine syndrome (FAS) is a progressive congestive ischaemic myelopathy caused by a spinal dural arteriovenous fistula (SDAVF) [1]. Accounting for approximately 70% of spinal vascular malformations, SDAVFs predominantly affect middle-aged and elderly men [1]. Despite its distinct pathophysiology, FAS remains a challenging diagnostic entity, with a median delay from symptom onset to correct diagnosis exceeding 15 months [2]. Initial misdiagnosis as transverse myelitis or idiopathic spinal cord infarction is common [2]. We present a case illustrating the diagnostic challenge of FAS resulting from overlooked perimedullary flow voids on initial magnetic resonance imaging (MRI).

## 2. Case Presentation

A 66-year-old man with a history of arterial hypertension, atrial fibrillation (on anticoagulation therapy with dabigatran), hypercholesterolaemia, and benign prostatic hyperplasia was admitted to our tertiary neurology department in February 2026 because of progressive paraparesis, sensory loss below Th12, and sphincter dysfunction. He had previously undergone left total knee arthroplasty (December 2024) without complications, followed by revision surgery in February 2025, performed under spinal anaesthesia. The spinal anaesthesia was administered atraumatically in the sitting position at the L3–L4 interspace using a 27-gauge pencil-point needle, with 3 mL of 0.5% hyperbaric bupivacaine and 20 µg fentanyl, achieving a sensory block to the Th10 dermatome. Clear cerebrospinal fluid was obtained without paraesthesiae during needle placement, and intraoperative haemodynamics remained stable, with no significant hypotension. Dabigatran had been discontinued 48 h before surgery and resumed 48 h afterwards. Notably, the patient had no neurological symptoms prior to the revision arthroplasty—specifically no gait disturbance, sensory symptoms, urinary dysfunction, or exercise-related leg weakness. Shortly postoperatively, he developed insidious, progressive lower-limb weakness and sensory disturbances.

**Figure 1:**
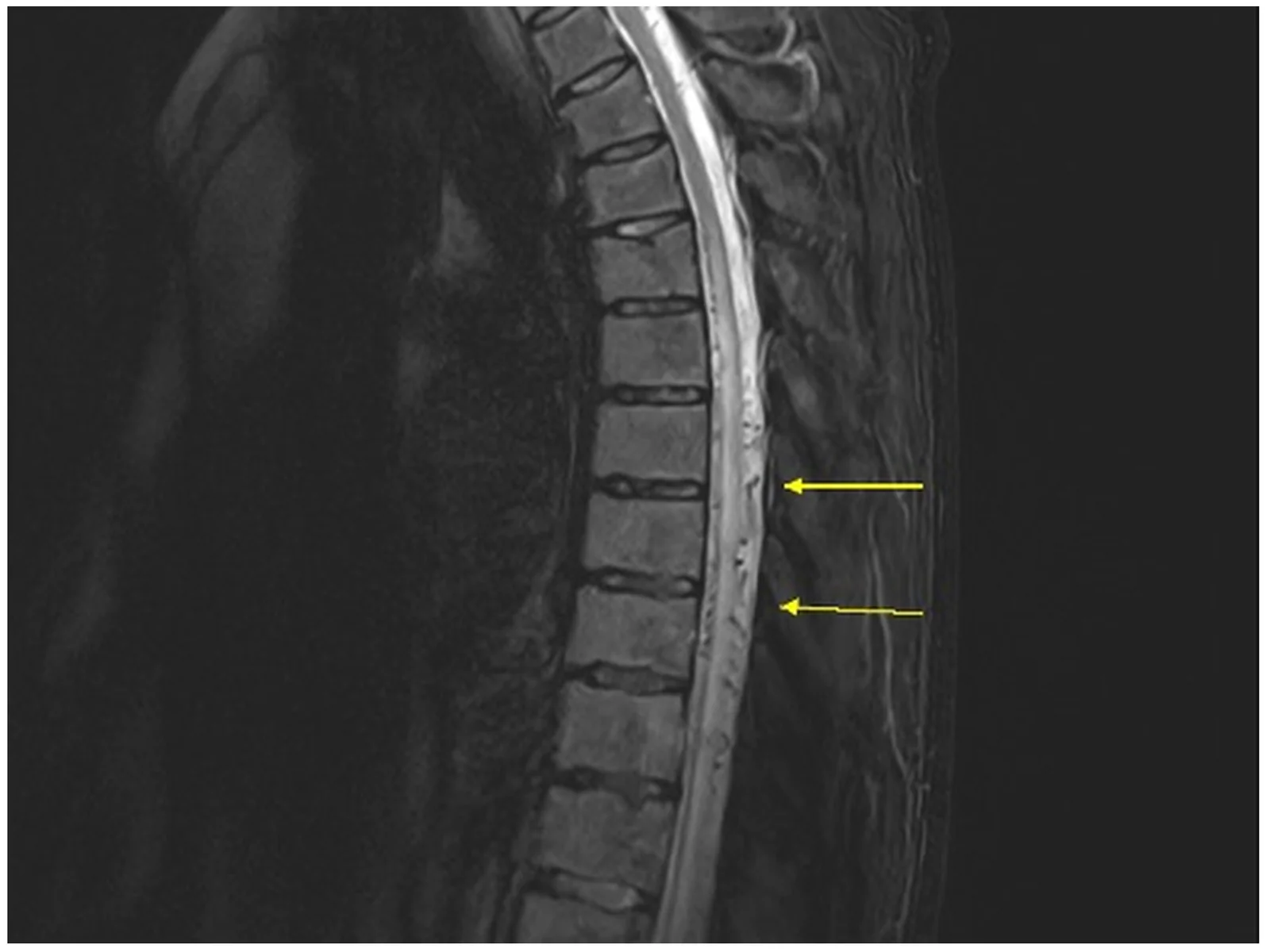
Initial thoracic spine MRI performed at the referring institution (June 2025). Sagittal T2-weighted image demonstrating diffuse intramedullary hyperintensity extending from Th6 to the conus medullaris. Perimedullary flow voids (arrows) were present but were not described in the original radiological report.

In June 2025, he was hospitalised at a regional neurology department. Thoracic spine MRI demonstrated diffuse T2 signal hyperintensity extending from the Th6 level to the conus medullaris, involving almost the entire cord cross-section in the lower segments—findings considered consistent with spinal cord infarction or transverse myelitis (Figure 1).

Perimedullary flow voids were in fact already present on these images but were not described in the original radiological report; consequently, SDAVF was not considered in the differential diagnosis at that time. A diagnostic lumbar puncture was performed, yielding cerebrospinal fluid with normal parameters (cell count: 3 cells/µL; protein: 32 mg/dL), followed by high-dose intravenous corticosteroid therapy, after which a marked worsening of neurological deficits (worsening lower-limb weakness and progression of gait disturbance) was observed within 24–48 h. A diagnosis of ischaemic spinal cord injury was established, and the patient was transferred to the Department of Neurological Rehabilitation for a period of 10 weeks. At discharge, he was ambulatory with a walking stick and independent in activities of daily living. Following discharge, his neurological condition deteriorated again, and he was admitted to our centre in February 2026.

## 3. Diagnostic Assessment

Neurological examination on admission revealed a pyramidal syndrome with paraparesis (lower-limb strength 3/5 on the Lovett scale), gait disturbance (mobilising with forearm crutches), a sensory deficit with a level at the Th12/L1 dermatome, and sphincter dysfunction characterised by urinary urgency with intermittent incontinence. The modified Aminoff-Logue Scale score was gait grade 4, micturition grade 2, and defecation grade 0 (G4, M2, D0).

Laboratory investigations were performed: serum vitamin B12, folate, and homocysteine levels were within normal limits; rheumatological, haematological, and muscle-disease panels and tumour markers showed no abnormalities; serological screening for syphilis and HIV was negative; and testing for anti-AQP4 and anti-MOG antibodies returned negative results. Bladder ultrasound demonstrated approximately 250 mL of post-void residual urine.

**Figure 2:**
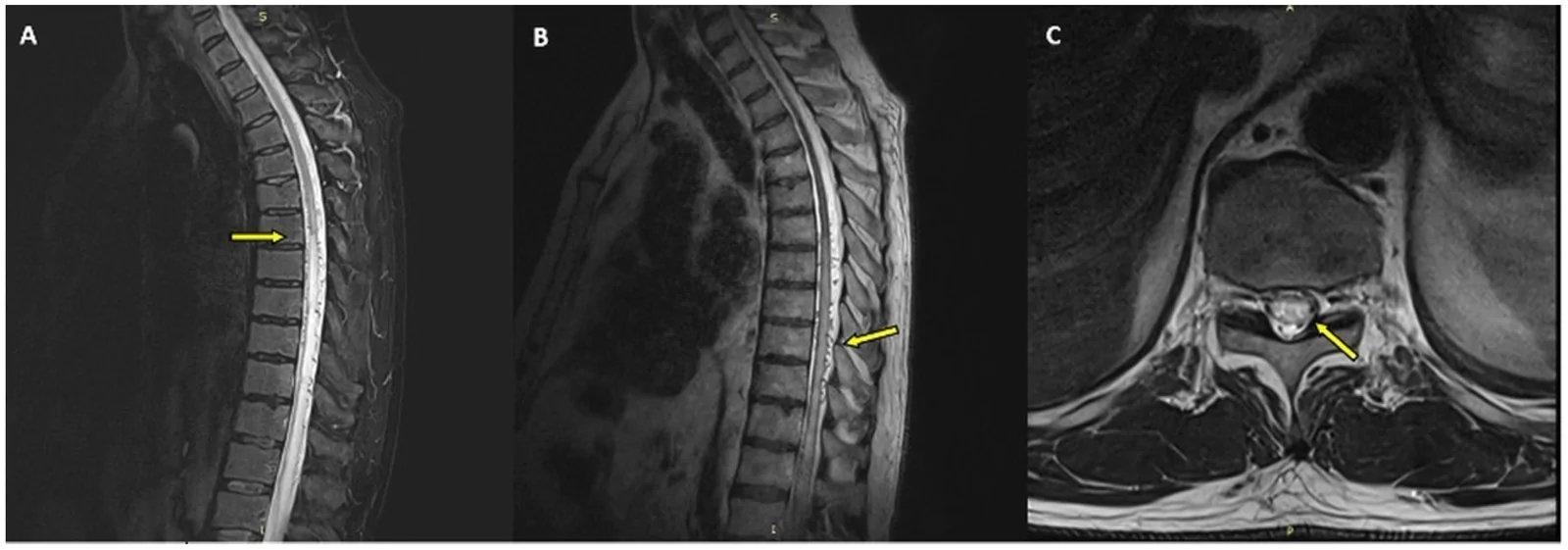
Thoracic spine MRI (3 Tesla, February 2026). T2-weighted image (TSE, STIR, sagittal)—(**A**) increased signal intensity from the Th7 level to the conus medullaris (arrow); (**B**) T2-weighted image (SPACE, sagittal, isotropic)—flow voids representing dilated perimedullary venous vessels (arrow). (**C**) T2-weighted axial image (TSE, Th11/12 level)—increased signal intensity involving almost the entire cord cross-section with punctate foci of contrast enhancement (arrow).

A repeat 3T MRI of the thoracic and lumbosacral spine demonstrated increased T2/FLAIR signal intensity and enlargement of the spinal cord from Th7 to the conus medullaris, with changes involving the entire cord cross-section, and punctate foci of contrast enhancement, together with prominent serpentine perimedullary flow voids indicating dilated pial veins (Figure 2).

**Figure 3:**
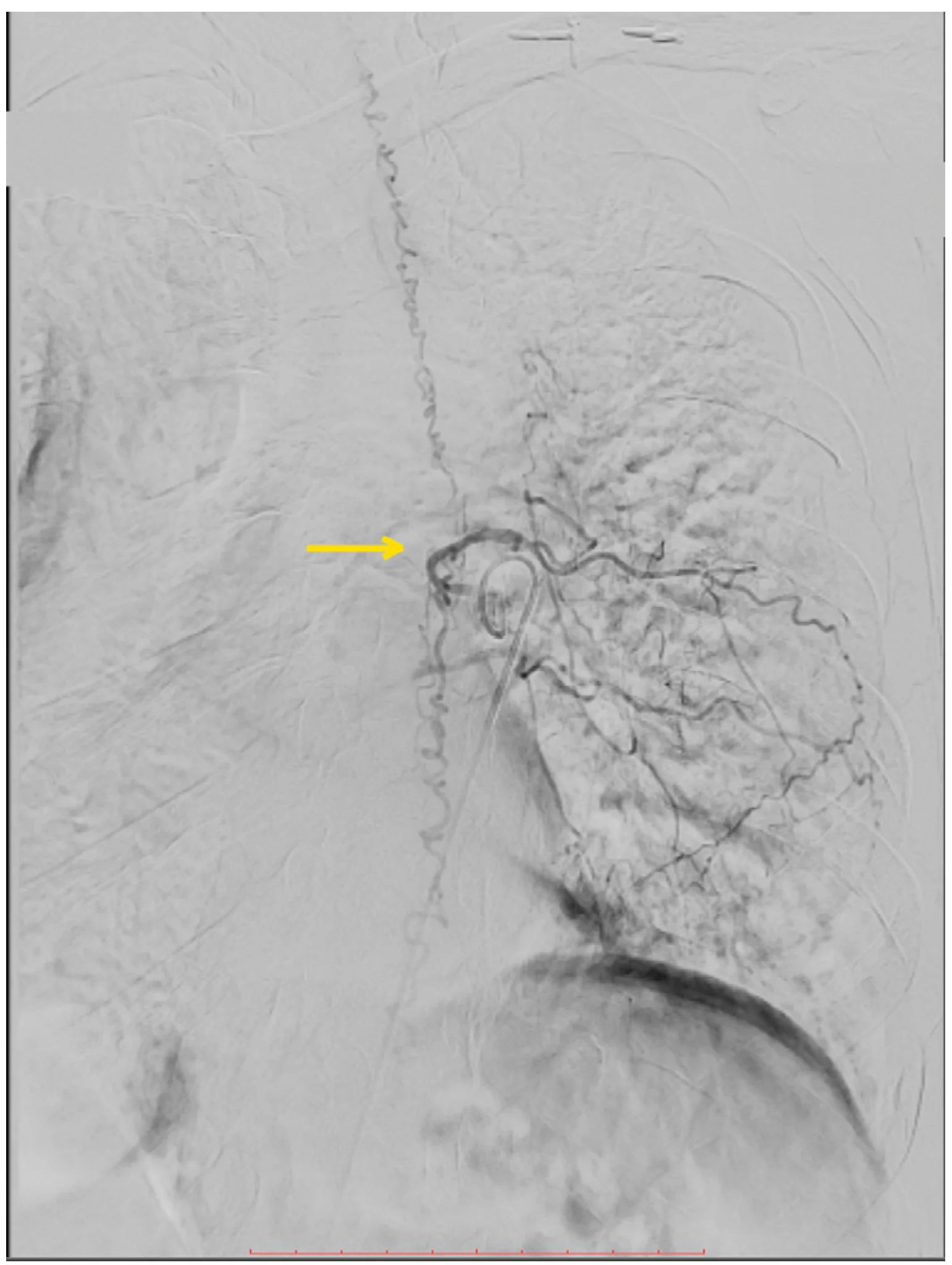
Digital subtraction angiography (DSA) demonstrating the spinal dural arteriovenous fistula at the Th5–Th6 level (arrow), with dilated perimedullary venous plexus (pre-embolisation).

Based on these findings, a spinal dural arteriovenous fistula was suspected. Digital subtraction angiography (DSA) was performed via right common femoral artery puncture, confirming the fistula at the Th5–Th6 level, supplied by a radiculomeningeal branch arising from the left intercostal artery, with retrograde drainage through a radiculomedullary vein and filling of the perimedullary coronal venous plexus (Figure 3). A diagnosis of Foix–Alajouanine syndrome was established.

### 3.1. Therapeutic Intervention

**Figure 4:**
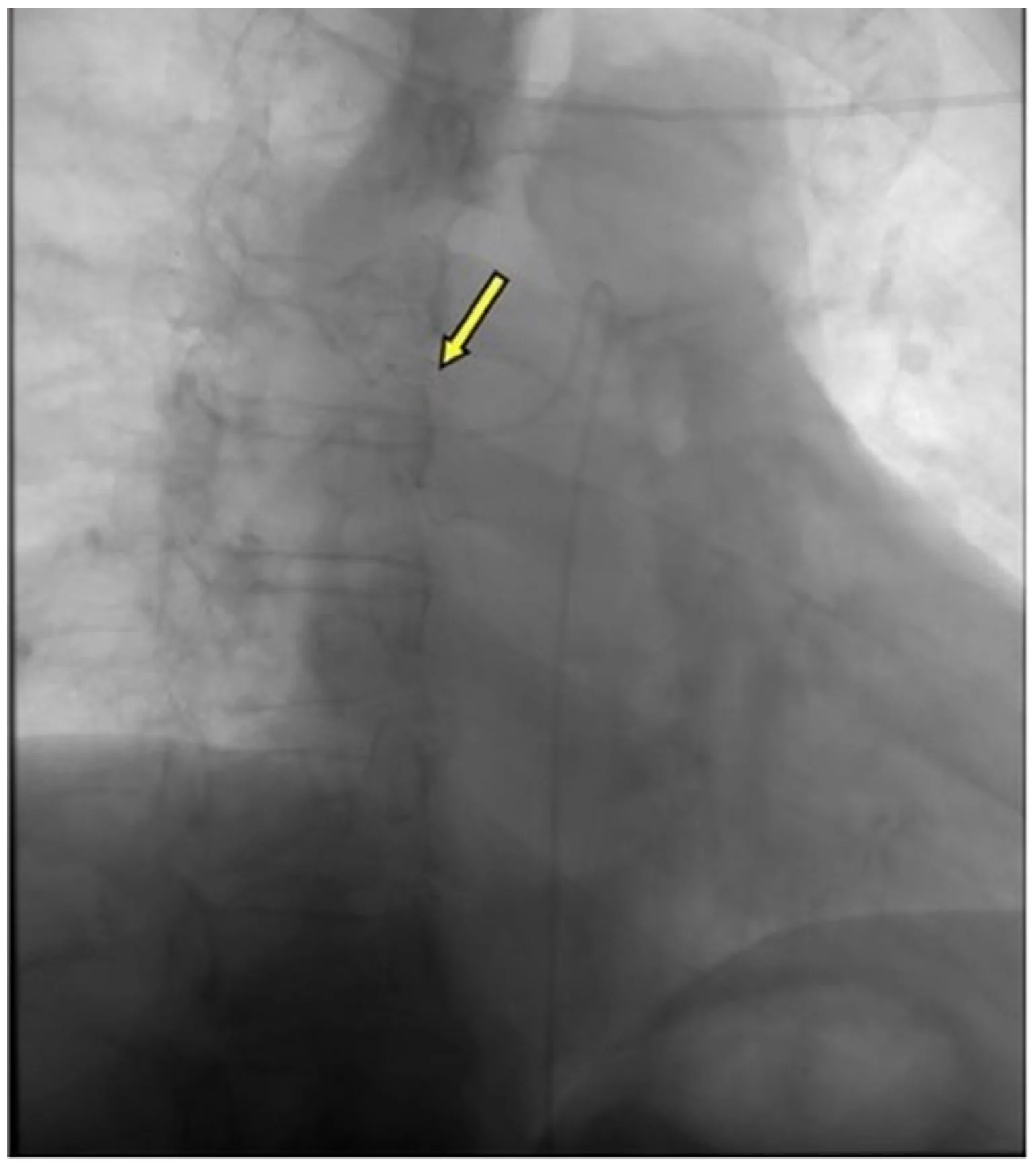
Post-embolisation angiogram demonstrating occlusion of the arteriovenous fistula (arrow).

The patient was qualified for endovascular treatment following multidisciplinary consultation with the neurosurgical team and interventional radiologist. Embolisation of the fistula was performed via a transarterial approach through the Th5 and Th6 intercostal arteries using a Sonic microcatheter, with Onyx (ethylene-vinyl alcohol copolymer) as the embolic material; the post-embolisation angiogram is shown in Figure 4. The postoperative course was uneventful.

### 3.2. Follow-Up and Outcomes

At discharge, the patient reported mild subjective improvement in lower-limb pain, although gait disturbance remained unchanged, and inpatient neurological rehabilitation was initiated. One month after the procedure, he reported neurological improvement, including reduction in lower-limb pain and paraesthesiae and mild improvement in gait. At the most recent follow-up, approximately five months after embolisation, he reported subjective improvement in bladder symptoms and partial reduction in lower-limb pain; however, he still requires a walking stick for ambulation. A follow-up MRI performed in June 2026 showed findings comparable to the pre-treatment study; a further follow-up MRI is planned, after which a decision regarding any additional intervention will be made. A summary of the clinical timeline is provided in Table 1.

## 4. Discussion

Foix–Alajouanine syndrome (FAS) is a progressive ischaemic myelopathy caused by a spinal dural arteriovenous fistula (SDAVF). It predominantly affects men, who account for approximately 72% of cases, with a male-to-female ratio of 5:1, and a mean age of onset of approximately 55 years [1,3]. SDAVFs are the most common spinal vascular malformations (approximately 1/100,000/year), causing progressive myelopathy [4,5]. Although SDAVFs may occur at any level of the spinal cord, more than 80% of lesions are located between Th5 and L2 [3]. These are low-flow fistulas that drain intradurally into the coronal venous plexus, which becomes tortuous and elongated over time. Due to the absence of valves between the coronal venous plexuses and the intramedullary veins, venous congestion develops—the presence of the fistula leads to increased venous pressure, a reduced arteriovenous pressure gradient, impaired spinal cord perfusion, and ultimately tissue necrosis [2,3,5].

Initial clinical manifestations are often nonspecific and include progressive lower limb weakness, limb pain, numbness, paraesthesiae, and sphincter dysfunction [2,5]. As the disease progresses, the clinical picture becomes more characteristic—the main symptoms are lower limb muscle weakness (84.8%), bladder dysfunction (69.6%), sensory deficits (65.2%), and gait disturbance (65.2%) [1]. Motor symptoms may be flaccid or spastic in nature; sensory disturbances include numbness and paraesthesiae of the distal lower extremities and perineal region, as well as back pain; sphincter dysfunction manifests as urinary hesitancy, faecal incontinence, erectile dysfunction, and involuntary ejaculation during physical exertion [6,7]. Symptom progression is typically gradual; however, acute onset over minutes or hours may also occur. Acute deterioration has been associated with physical exertion, prolonged standing, singing [8], corticosteroid administration [9], and lumbar puncture [10]. Previously reported triggers of acute deterioration in SDAVF, together with the proposed role of spinal anaesthesia, are summarised in Table 2.

The key diagnostic tool is spinal cord MRI, which typically demonstrates intramedullary T2 hyperintensities corresponding to vasogenic oedema, as well as dilated, tortuous, serpentine perimedullary vessels appearing as flow voids. Digital subtraction angiography (DSA) remains the gold standard for the definitive diagnosis of SDAVF [11,12].

The initial differential diagnosis included both inflammatory longitudinally extensive transverse myelitis (LETM) and ischaemic spinal cord injury, as SDAVF may closely mimic both conditions clinically and radiologically [13,14]. However, the differential diagnosis of LETM is considerably broader and includes compressive, neoplastic, infectious, metabolic, nutritional, and inflammatory disorders [12]. In the present case, contrast-enhanced MRI excluded compressive and neoplastic lesions, while normal cerebrospinal fluid findings and negative serological tests, including those for syphilis and HIV, made an infectious aetiology unlikely. Metabolic and nutritional aetiologies were considered unlikely on the basis of normal serum vitamin B12, folate, and homocysteine levels. An inflammatory or demyelinating aetiology, including neuromyelitis optica spectrum disorder and MOG-associated disease, was considered less likely based on the overall clinical, laboratory and imaging findings, including negative anti-AQP4 and anti-MOG antibody tests and the absence of clinical improvement following corticosteroid therapy. The acute-phase MRI findings were equivocal, as diffuse T2 signal hyperintensity within the spinal cord may represent venous congestive myelopathy in SDAVF, transverse myelitis, or spinal cord infarction, particularly in the early stages. Neurological deterioration following corticosteroid treatment initially reinforced the diagnosis of spinal cord ischaemia; however, in retrospect, this paradoxical worsening is a well-documented feature of SDAVF and should prompt further evaluation for an underlying spinal vascular malformation [9]. Ultimately, the identification of characteristic perimedullary flow voids on MRI, followed by confirmation of SDAVF by DSA, established the correct diagnosis [11,12].

Although the temporal association with spinal anaesthesia initially raised concern for a procedure-related complication, alternative perioperative causes of acute myelopathy were considered and deemed unlikely. Procedure-related neural injury was unlikely, as spinal anaesthesia was performed atraumatically at the L3–L4 interspace, well below the conus medullaris, with no paraesthesiae during needle placement. Perioperative spinal cord ischaemia due to haemodynamic compromise was considered unlikely because intraoperative haemodynamic parameters remained stable without significant hypotension. The absence of acute back pain or immediate postoperative neurological deficits argued against an epidural haematoma. Furthermore, dabigatran had been discontinued 48 h before surgery and resumed 48 h after the procedure. Finally, the gradual progression of symptoms over several months was far more consistent with venous congestive myelopathy due to SDAVF than with an acute perioperative vascular or mechanical complication [1,3,6].

The median time from symptom onset to correct diagnosis is approximately 15 months [6], which is consistent with the clinical course of the presented case. Diagnostic delay is associated with poorer neurological outcomes due to irreversible spinal cord injury resulting from chronic venous congestion [14,15].

Symptom onset in this patient occurred in temporal association with spinal anaesthesia performed for revision knee arthroplasty, and we suspect the fistula was already present beforehand. A plausible mechanism, analogous to that described after lumbar puncture, is a sudden decrease in cerebrospinal fluid pressure: under normal conditions this pressure provides a counter-pressure that limits distension of the perimedullary venous plexus, and in a spinal cord already compromised by venous hypertension from an SDAVF, an abrupt reduction may permit engorgement of the already congested perimedullary veins, aggravating venous congestion and precipitating clinical deterioration [4,10]. This association is temporal only, however, and causation cannot be established; it is therefore presented as a hypothesis rather than a proven mechanism.

A critical diagnostic clue in the presented case was the worsening of neurological status following corticosteroid administration—a phenomenon well documented in SDAVF [9]—which, in retrospect, should have prompted further vascular investigation. The mechanism of steroid-induced deterioration is attributable to sodium and fluid retention mediated by aldosterone, leading to increased venous hydrostatic pressure, further impairment of spinal venous drainage, and ultimately venous infarction [16]. It should also be noted that the lumbar puncture performed during the initial hospitalisation may have constituted an additional factor exacerbating venous congestion—through a sudden decrease in cerebrospinal fluid pressure and rapid engorgement of meningeal veins [10]—a phenomenon previously described in the context of SDAVF.

This case also underscores an important radiological teaching point. Perimedullary flow voids, although a characteristic imaging feature of SDAVF, may be subtle and are readily overlooked on conventional T2-weighted MRI, particularly when clinical suspicion is low [17]. Advanced MRI techniques, including three-dimensional heavily T2-weighted sequences, may improve their detection [18]. Susceptibility-weighted imaging and contrast-enhanced MR angiography may provide additional diagnostic value in equivocal cases. When longitudinally extensive myelopathy of uncertain aetiology is encountered, particularly in patients with a clinical course atypical for inflammatory or ischaemic disease or worsening after corticosteroid therapy, the initial MRI should be carefully re-reviewed for perimedullary flow voids, and dedicated spinal vascular imaging should be considered. It should also be noted that not all spinal dural arteriovenous fistulas produce prominent perimedullary flow voids; in particular, some low lumbosacral fistulas may lack conspicuous flow voids, and their absence does not exclude the diagnosis [19]. Therefore, spinal angiography should be considered in any patient with a suspected fistula, even when flow voids are not prominent on MRI.

## 5. Conclusions

This case highlights that perimedullary flow voids, although a characteristic feature of SDAVF, are readily overlooked on initial imaging, leading to misdiagnosis and potentially harmful interventions. In any patient with unexplained, longitudinally extensive myelopathy, the initial MRI should be systematically re-examined for vascular flow voids, and neurological deterioration following lumbar puncture, corticosteroids, or neuraxial procedures should raise suspicion of an underlying spinal vascular malformation and prompt dedicated vascular imaging. Early diagnosis and treatment are essential to prevent irreversible spinal cord injury.

## Figures and Tables

**Table 1:** Clinical timeline.

Date	Event	Clinical Status/Findings
December 2024	Left total knee arthroplasty	No neurological symptoms
February 2025	Revision knee arthroplasty under spinal anaesthesia	Onset of progressive lower limb weakness and sensory disturbances; no prior neurological symptoms
June 2025	Referring hospital: MRI, lumbar puncture, IV corticosteroids	MRI: T2 hyperintensity Th6–conus; flow voids present but unreported. CSF normal. Deterioration within 24–48 h of steroids
June–August 2025	10-week rehabilitation	Ambulatory with a walking stick
February 2026	Our centre: repeat MRI, DSA, embolisation	SDAVF at Th5–Th6; embolisation with Onyx (Sonic microcatheter). FAS diagnosed
~1 month later	Follow-up	Reduced pain and paraesthesiae, mild gait improvement
June/July 2026	Most recent follow-up	Control MRI comparable to previous study; subjective improvement in bladder symptoms and partial pain reduction; still uses a walking stick; further follow-up MRI planned

CSF, cerebrospinal fluid; DSA, digital subtraction angiography; FAS, Foix–Alajouanine syndrome; IV, intravenous; MRI, magnetic resonance imaging; SDAVF, spinal dural arteriovenous fistula.

**Table 2:** Reported triggers of acute neurological deterioration in spinal dural arteriovenous fistula, with the proposed mechanism for spinal anaesthesia shown in the context of established precipitants.

Trigger	Proposed Mechanism	Ref.
Lumbar puncture	Sudden decrease in cerebrospinal fluid pressure leading to perimedullary venous engorgement	[10]
Corticosteroid administration	Sodium and fluid retention increasing venous hydrostatic pressure	[9]
Physical exertion	Increased venous return and spinal venous pressure	[8]
Prolonged standing	Orthostatic increase in spinal venous pressure	[8]
Singing/Valsalva manoeuvre	Raised intrathoracic and spinal venous pressure	[8]
Spinal anaesthesia (present case)	Sudden decrease in cerebrospinal fluid pressure leading to perimedullary venous engorgement, analogous to lumbar puncture	—

The mechanism proposed for spinal anaesthesia is analogous to that established for lumbar puncture.

## Data Availability

All data generated or analysed during this study are included in this published article.
